# Diagnostic value of transmural perfusion ratio derived from dynamic CT-based myocardial perfusion imaging for the detection of haemodynamically relevant coronary artery stenosis

**DOI:** 10.1007/s00330-016-4567-0

**Published:** 2016-10-04

**Authors:** Adriaan Coenen, Marisa M. Lubbers, Akira Kurata, Atsushi Kono, Admir Dedic, Raluca G. Chelu, Marcel L. Dijkshoorn, Alexia Rossi, Robert-Jan M. van Geuns, Koen Nieman

**Affiliations:** 1 Department of Radiology, Erasmus University Medical Center, Rotterdam, the Netherlands; 2 Department of Cardiology, Erasmus University Medical Center, Rotterdam, the Netherlands; 3 NIHR Cardiovascular Biomedical Research Unit at Barts, William Harvey Research Institute, Barts and The London School of Medicine and Dentistry, Queen Mary University of London & Department of Cardiology, Barts Health NHS Trust, London, UK

**Keywords:** Coronary artery disease, Perfusion, Fractional flow reserve, myocardial, Tomography, X-ray computed

## Abstract

**Objectives:**

To investigate the additional value of transmural perfusion ratio (TPR) in dynamic CT myocardial perfusion imaging for detection of haemodynamically significant coronary artery disease compared with fractional flow reserve (FFR).

**Methods:**

Subjects with suspected or known coronary artery disease were prospectively included and underwent a CT-MPI examination. From the CT-MPI time-point data absolute myocardial blood flow (MBF) values were temporally resolved using a hybrid deconvolution model. An absolute MBF value was measured in the suspected perfusion defect. TPR was defined as the ratio between the subendocardial and subepicardial MBF. TPR and MBF results were compared with invasive FFR using a threshold of 0.80.

**Results:**

Forty-three patients and 94 territories were analysed. The area under the receiver operator curve was larger for MBF (0.78) compared with TPR (0.65, *P* = 0.026). No significant differences were found in diagnostic classification between MBF and TPR with a territory-based accuracy of 77 % (67-86 %) for MBF compared with 70 % (60-81 %) for TPR. Combined MBF and TPR classification did not improve the diagnostic classification.

**Conclusions:**

Dynamic CT-MPI-based transmural perfusion ratio predicts haemodynamically significant coronary artery disease. However, diagnostic performance of dynamic CT-MPI-derived TPR is inferior to quantified MBF and has limited incremental value.

***Key Points*:**

• *The transmural perfusion ratio from dynamic CT-MPI predicts functional obstructive coronary artery disease*

• *Performance of the transmural perfusion ratio is inferior to quantified myocardial blood flow*

• *The incremental value of the transmural perfusion ratio is limited*

## Introduction

Dynamic computed tomography myocardial perfusion imaging (CT-MPI) is based on sequential scanning of the myocardium during the first pass of a contrast bolus. The dynamic imaging of the contrast medium allows for a non-invasive quantification of myocardial blood flow (MBF), until now mainly performed with either magnetic resonance imaging or positron emission tomography [1, 2]. With recent developments in CT scanners this technique also became available for CT imaging [3, 4]. The diagnostic performance of CT-MPI compared with fractional flow reserve is good [5–7]. However, possible underestimation of absolute MBF values by CT-MPI is a potential concern [4, 8].

Reduced myocardial perfusion due to coronary artery disease (CAD) tends to be more pronounced in the subendocardium [9]. The high spatial resolution of CT allows for distinguishing the subendocardium and subepicardium. A method to utilise the susceptibility of the subendocardium for ischaemia is the transmural perfusion ratio (TPR) [10]. TPR is the ratio between subendocardium and subepicardium perfusion. As TPR is a relative index we hypothesised it would be less influenced by lower absolute MBF values and improve the diagnostic performance of CT-MPI.

In this study TPR and MBF based on dynamic CT-MPI are investigated individually and in combination, and compared with the invasive fractional flow reserve (FFR).

## Methods

### Study design

The local institutional review board approved this prospective study. Written informed consent was obtained from all patients. This study included cases from a previous study investigating the diagnostic performance of CT-MPI [7]. Patients with suspected or known CAD referred for invasive angiography were prospectively recruited. Included patients underwent a dynamic CT-MPI examination 1-14 days before invasive angiography.

This study was designed to investigate the ability of CT-MPI to detect ischaemia; therefore only territories with an FFR measurement in the associated coronary artery were included. Territories associated with a (sub)total occluded coronary artery where no FFR measurement could be performed were not included in the analysis.

### Recruitment and population

Patients with suspected or known coronary artery disease referred for invasive angiography were recruited in the time period December 2010 until December 2014. Exclusion criteria were younger than 40 years old, impaired renal function (serum creatinine >120 μmol/l), possible pregnancy or breast feeding, body weight over 120 kg, use of clopidogrel, contra-indications for iodine contrast medium, or contra-indications for adenosine.

### CT-MPI acquisition

All patients were requested to refrain from caffeine intake 24 h prior to the examination. In both arms 18-gauge cannulas were inserted in the antecubital veins. Blood pressure and ECG were monitored during the examination. Forty patients were scanned with a second-generation dual-source CT scanner and three patients with a third-generation dual-source CT scanner (SOMATOM Definition Flash and SOMATOM Force, Siemens Medical Solutions, Forchheim, Germany). Adenosine was infused at a rate of 140 μg/kg/min. CT-MPI acquisition was started 3 min after start of adenosine infusion.

The acquisition protocol consisted of coronary CT angiography, a non-contrast scan and the dynamic CT-MPI scan. The non-contrast scan was acquired during end systole and served for planning of the CT-MPI. Before the CT-MPI acquisition all patients received sublingual nitroglycerine. Intravenous beta blockers were used in patients with high heart rates prior to the coronary CT angiography, but very infrequently (*N* = 3) as these potentially affect the CT-MPI performance. After 3 min of adenosine infusion, 50 ml of contrast medium (Ultravist, 370 mgI/ml; Bayer, Berlin, Germany) was injected at 6 ml/s, followed by a saline bolus of 40 ml. All CT-MPI studies were made with an axial scan mode at 250 ms after the R wave (end systolic). To sufficiently cover the left ventricle the myocardial acquisition was performed in alternating cranial and caudal table positions (shuttle mode), acquiring two slightly overlapping data sets [3]. CT-MPI acquisition was started 5 s after the start of the contrast medium injection. Patients were asked to hold their breath during the entire dynamic CT-MPI acquisition (30-35 s). The number of time points acquired varied per patient depending on the heart rate: 1 patient had 9 cranial and caudal time points, 11 patients had 10, 6 patients had 11, 12 patients had 12, 9 patients had 13, and 4 patients had 14 time points.

The second-generation dual-source CT scanner used the following scan parameters: collimation 2 × 64 × 0.6-mm detector collimation with flying z-spot technique [11], gantry rotation time 280 ms, temporal resolution 75 ms, tube voltage/current 100 kVp/300 mAs and shuttle-mode coverage 73 mm.

The third-generation dual-source CT scanner used the following scan parameters: collimation 2 × 96 × 0.6-mm detector collimation with flying z-spot technique, gantry rotation time 250 ms and temporal resolution 66 ms; Care-Kv [12] was used with reference settings for tube voltage/current: 80 kVp/300 mAs and shuttle-mode coverage 102 mm.

### Post processing

The CT-MPI images were reconstructed using a dedicated kernel for reduction of iodine beam-hardening artefacts (b23f, Qr36) and transferred to a CT-MPI analysis software package (Volume Perfusion CT body, Syngo Somaris/7; Siemens, Germany). Motion correction was applied if necessary to correct for breathing displacement. The motion correction algorithm uses a time point selected by the user (with contrast in the left and right ventricle and smooth connection between the cranial and caudal section) and then registers the other time points to the selected time point using non-rigid registration. The left ventricle is segmented by combining thresholding and peak enhancement [13]. The change of attenuation in the myocardium over time was computed by creating time-attenuation curves (TACs). For quantification of the MBF the influx of contrast bolus was measured with an arterial input function (AIF). The AIF was measured by placement of an ROI in the descending aorta in the CT-MPI images. Precision of the AIF was increased by including both the cranial and caudal sections (double sampling). For quantification of the MBF the myocardial TACs were coupled with the AIF using a hybrid deconvolution model. The model generates a perfusion model curve based on the change in attenuation using a simplified impulse residue function for modelling the interaction between the intra- and extracellular compartments. The MBF was computed on a per voxel basis by dividing the maximal slope of the model curve for the myocardial tissue by the maximum AIF [3, 4, 14]. MBF data sets were reconstructed with a 512 × 512 matrix resulting in a pixel size of 0.35 × 0.35 mm and were reconstructed as a stack of colour-coded maps with a slice thickness of 3 mm and an increment of 1.5 mm.

### Image analysis

MBF and TPR were individually evaluated by readers with previous experience in dynamic CT-MPI examinations. Both readers were provided with the colour-coded CT-MPI data sets. For each patient a list of vessels investigated by FFR was provided. To ensure correct territory-vessel correspondence left or right coronary dominance was provided for each patient. All readers were asked to measure MBF or TPR value corresponding to the vessel where the FFR measurements were performed. Each independent reader was blinded to all other medical information.

Within the MBF short axis slice interpreted as representing the myocardium dependent on the vessel in which the FFR was made, a freehand ROI was placed surrounding the suspected perfusion defect (Syngo Via 2.0, Siemens AG, Germany). The freehand ROI had a minimal area of 50 mm^2^. Careful considerations were made to prevent inclusion of artefacts in the ROI.

**Fig. 1:**
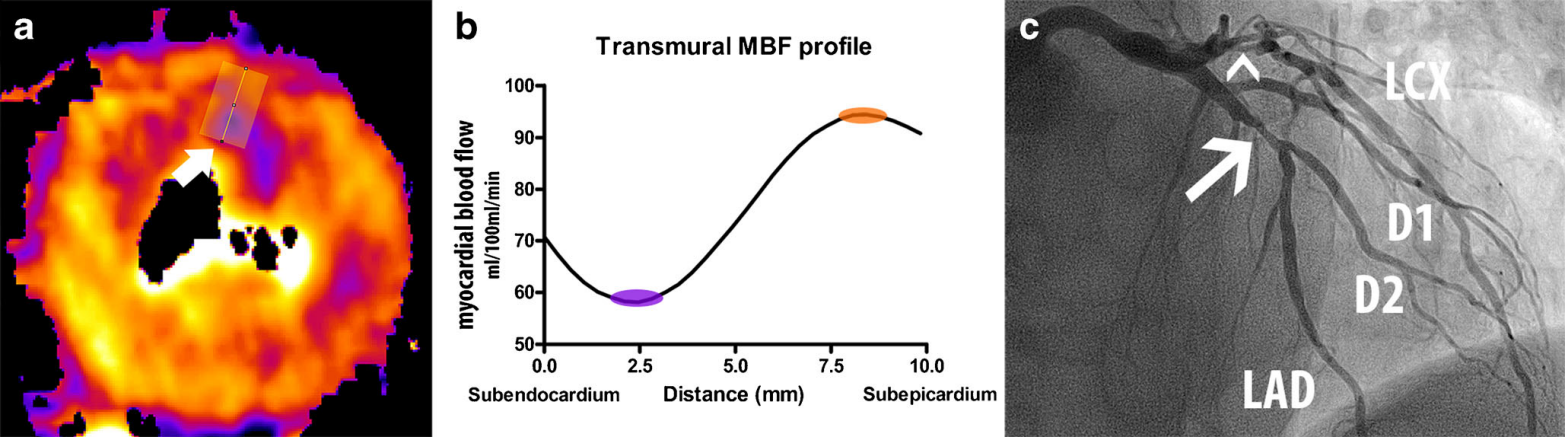
TPR case example: 65-year-old male presenting after exertional collapse. **a** Short-axis CT-MPI image with the transmural perfusion line placed in the anterior-lateral segment (*white arrow*). **b** The transmural MBF profile. The subendocardial MBF was 58 ml/100 ml/min (*purple marker*) and 91 ml/100 ml/min in the subepicardium (*orange marker*). The TPR was 0.64 (58/91) and thus considered positive for ischaemia. **c** Invasive angiography showing a stenosis in the proximal LAD with an FFR of 0.69. A subtotal stenosis was directly stented in the LCX (*arrowhead*); as such no FFR measurement was performed. In panel **a** however a perfusion defect with a transmural perfusion ratio can be seen in the territory associated with the LCX. The RCA was normal with an FFR of 0.91. *RCA*: right coronary artery, *LAD*: left anterior descending artery, *LCX*: left circumflex artery, *TPR*: transmural perfusion ratio, *MBF*: myocardial blood flow, *FFR*: fractional flow reserve

For TPR the CT-MPI colour-coded maps were visually assessed to identify the slice most representative for a subendocardial/subepicardial ratio. The section of interest was loaded onto a dedicated image analysis application (ImageJ 1.48, National Institutes of Health, USA) [15]. To measure the transmural differences in the MBF a series of linear samples perpendicular to the myocardial surface was taken at 0.4-mm equal intervals (Fig. 1). The mean MBF values from the pixels under the line are projected in the transmural MBF profile curve. Care was taken not to sample too close to the LV lumen and epicardial border as the MBF absolute values are unreliable because of displacement artefacts. From the short-axis MBF image and the MBF profile curve the user selected the endocardial and epicardial positions. The TPR was calculated by dividing the subendocardial by the subepicardial MBF.

### Invasive angiography and fractional flow reserve

Invasive coronary angiography was performed according to local clinical standards. Prior to the invasive angiography intracoronary nitroglycerine was given, as is the standard in our centre. Invasive FFR was performed in all vessels with a visual stenosis grade between 30-90 % by invasive angiography. By protocol, an FFR pressure wire (PressureWire Aeris/Certus, St. Jude Medical, St. Paul, USA, or Prime/Combo Wire, Volcano, San Diego, CA, USA) was placed distal to the stenosis of interest, after which hyperaemia was induced by intravenous infusion of adenosine at 140 μg/kg/min. An invasive FFR ≤0.80 was considered haemodynamically significant.

### Statistics

Absolute variables are represented as total and percentage, continuous variables as mean and standard deviation (±). The mean values for MBF and TPR for normal and ischaemic territories were compared with an unpaired two-sided independent t-test. Pearson coefficient correlation was calculated for respectively MBF and TPR against invasive FFR. The receiver-operator characteristic (ROC) curves including the area under the curve (AUC) were presented for MBF and TPR. To investigate the combined diagnostic performance for MBF and TPR an ROC curve was also plotted for a new combined variable MBF multiplied by TPR (MBF × TPR). The optimal threshold for MBF and TPR diagnostic accuracy was calculated using the Youden index [16]. A sub-analysis was made for territories with an intermediate MBF between 50-100 ml/100 ml/min, as these represent territories with MBF values close to the diagnostic threshold [5, 7]. Diagnostic performance was evaluated as sensitivity, specificity, positive predictive value, negative predictive value and accuracy, with their corresponding 95 % confidence intervals (CI). The 95 % confidence intervals were corrected for within-subject clustering of data using variance adjustment [17]. MBF and TPR were displayed against each other with territories classified as normal or ischaemic. Inter-observer variability was determined for 72 (75 %) randomly selected territories by intra-class correlation coefficient for absolute MBF and TPR; diagnostic classification was compared using kappa statistics. Results were reported on a per-territory and per-patient basis and in accordance with the STARD initiative (Standard for Reporting Diagnostic accuracy) [18]. Most statistical analyses were made using SPSS (version 21, IBM Corp., Armonk NY, USA), while MedCalc (version 13.0; MedCalc Software, Ostend, Belgium) was used to compare the AUCs by using the method of DeLong et al. [19].

## Results

**Fig. 2:**
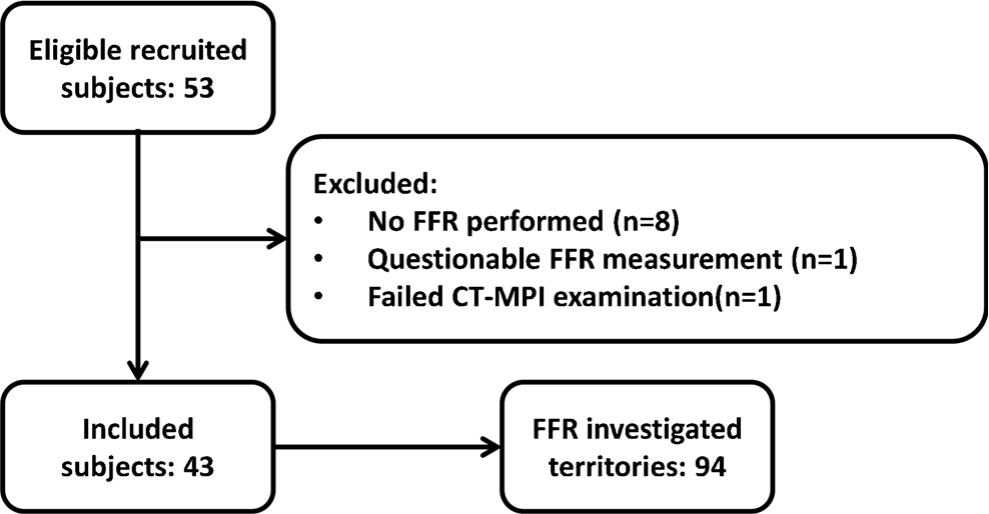
Inclusion flow chart

Fifty-three patients were recruited; 10 patients were excluded, 8 because of a lack of invasive FFR measurements (Fig. 2), resulting in a study population consisting of 43 patients, in whom 94 vessels were analysed by invasive FFR (Table 1). The mean FFR was 0.79 ± 0.17, with 48 vessels being considered haemodynamically significant with an FFR ≤0.80. The mean dose-length product for the CT-MPI acquisition was 640 ± 135 mGy-cm, resulting in an effective dose of 9.0 ± 1.9 mSv applying a conversion factor of 0.014 (Fig. 1).

**Table 1:** Patient characteristics

Number of patients, *n*	43
Age (years)	62.6 ± 8.7
Male gender, *n* (%)	36 (84)
Body mass index (kg/m^2^)*	20.1 ± 2.3
Body surface area (m^2)^*	2.0 ± 0.14
Cardiovascular risk factors, *n* (%)	
Hypertension	27 (63)
Dyslipidaemia	20 (47)
Diabetes	7 (16)
Family history of CAD	17 (40)
Smoking within the last year	10 (22)
Prior myocardial infarction, *n* (%)†	8 (19)
Prior PCI, *n* (%)†	5 (12)
Agatston coronary calcium score‡	628 (265-1450)
Heart rate during rest	63.4 ± 12.9
Heart rate during hyperaemic CT-MPI.	83.0 ± 13.7

Values are reported as mean and ± standard deviation or absolute number *n* and percentage (%). CAD, coronary artery disease; PCI, percutaneous coronary intervention

*In four patients length and weight data were not available

†Not in the vessel territories interrogated by invasive FFR

‡Represented in median and (quartiles)

**Fig. 3:**
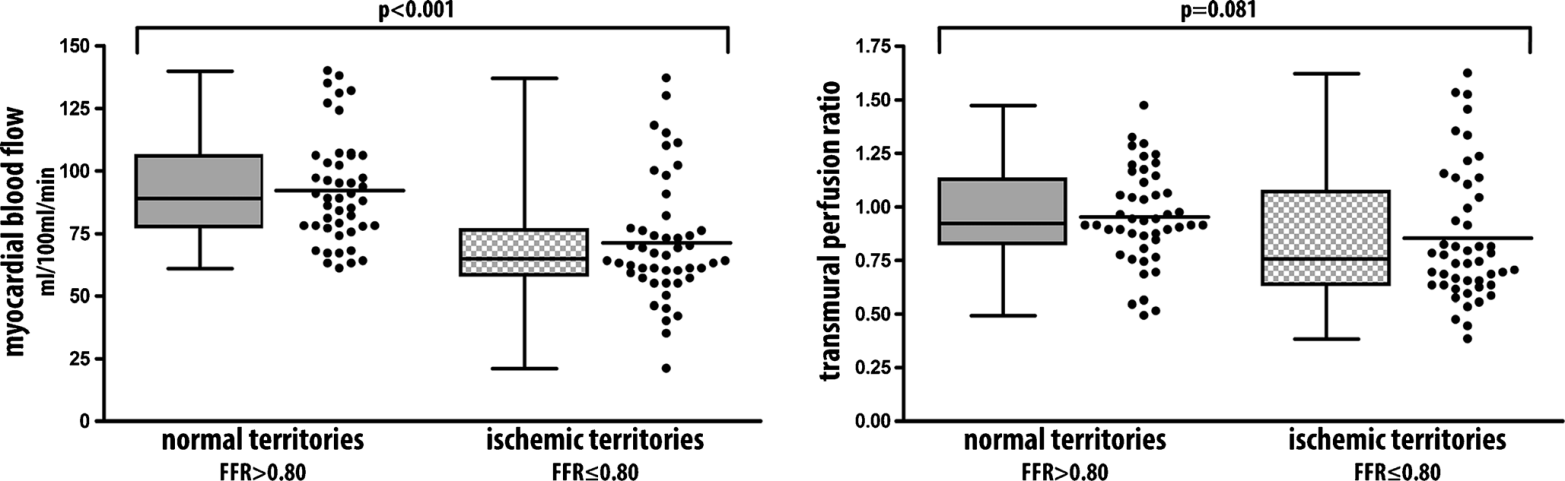
MBF and TPR: Median and mean myocardial blood flow and transmural perfusion ratio in 94 territories for normal (*N* = 46) and ischaemic (*N* = 48) territories. Normal territory defined as invasive FFR > 0.80, and ischaemic territories as FFR ≤ 0.80. *FFR*: fractional flow reserve

**Fig. 4:**
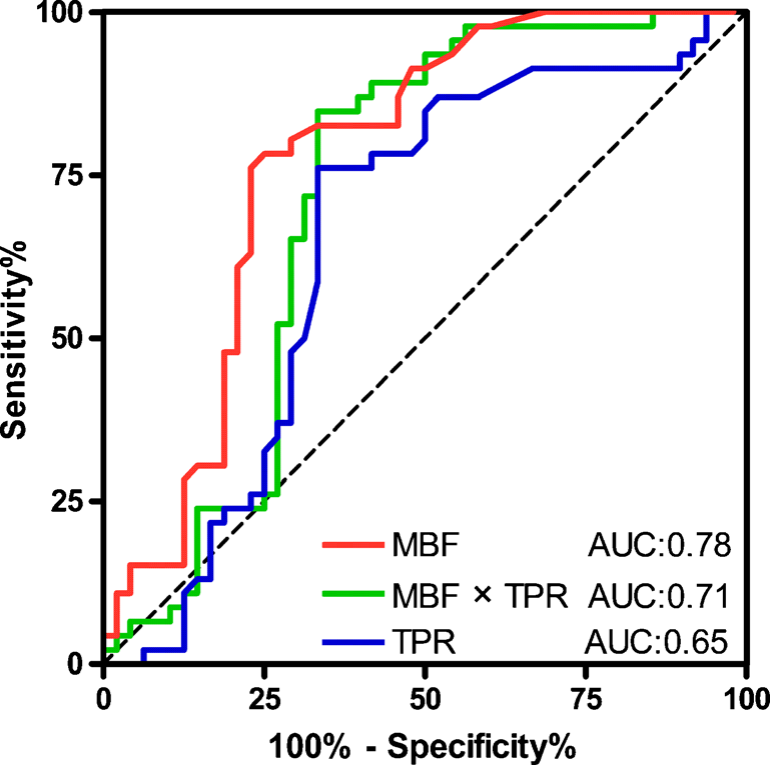
ROC: Receiver-operator curves for MBF and TPR validated against FFR using a threshold of 0.80 for haemodynamic significance. Area under the curve for MBF was 0.78 (95 % CI: 0.67-0.87), for TPR 0.65 (95 % CI: 0.53-0.77) and for MBF × TPR 0.71 (95 % CI: 0.60-0.82). The optimal diagnostic threshold was calculated at 76 ml/100 ml/min for MBF and 0.82 for TPR. *MBF*: myocardial blood flow, *TPR*: transmural perfusion ratio, *FFR*: fractional flow reserve, *CI*: confidence interval

The mean MBF for FFR confirmed ischaemic territories was 71.3 ± 24.3 ml/100 ml/min and for normal territories 92.2 ± 21.6 ml/100 ml/min (Fig. 3). The Pearson correlation coefficient was 0.55 for MBF directly compared with invasive FFR. The area under the curve (AUC) was 0.78 (Fig. 4). Optimal threshold for diagnostic classification was ≤76 ml/100 ml/min. The territory-based accuracy for MBF was 77 % (67-86 %) (Table 2).

**Table 2:** Diagnostic performance

All vessels (*n* = 94)	TP	FP	TN	FN	Sensitivity	Specificity	PPV	NPV	Accuracy
MBF	36	10	36	12	75 % (62-88 %)	78 % (66-90 %)	78 % (65-91 %)	75 % (63-87 %)	77 % (67-86 %)
TPR	31	11	35	17	65 % (50-79 %)	76 % (64-89 %)	74 % (59-88 %)	67 % (54-80 %)	70 % (60-81 %)
MBF 50-100 (*n* = 65)									
MBF	29	10	22	4	88 % (76-100 %)	69 % (53-85 %)	74 % (60-89 %)	85 % (71-98 %)	78 % (68-90 %)
TPR	24	8	24	9	73 % (54-89 %)	75 % (60-90 %)	75 % (59-91 %)	73 % (57-88 %)	74 % (62-86 %)

Diagnostic performance with invasive FFR using a threshold of ≤0.80. Territories with an MBF ≤76 ml/100 ml/min and TPR ≤0.82 were considered positive for ischaemia. A sub-analysis is made for territories with an intermediate MBF between 50 and 100 ml/100 ml/min. FFR: fractional flow reserve, MBF, myocardial blood flow, TPR: transmural perfusion ratio, PPV: positive predictive value, NPV: negative predictive value

The mean TPR for ischaemic territories was 0.85 ± 0.31 (Fig. 3). Pearson correlation between TPR and invasive FFR was 0.37. The AUC for TPR was 0.65 and significantly smaller than for MBF (*P* = 0.026). The optimal threshold for diagnostic classification was ≤0.82 (Fig. 4). The territory-based accuracy of TPR was 70 % (60-81 %) (Table 2).

**Fig. 5:**
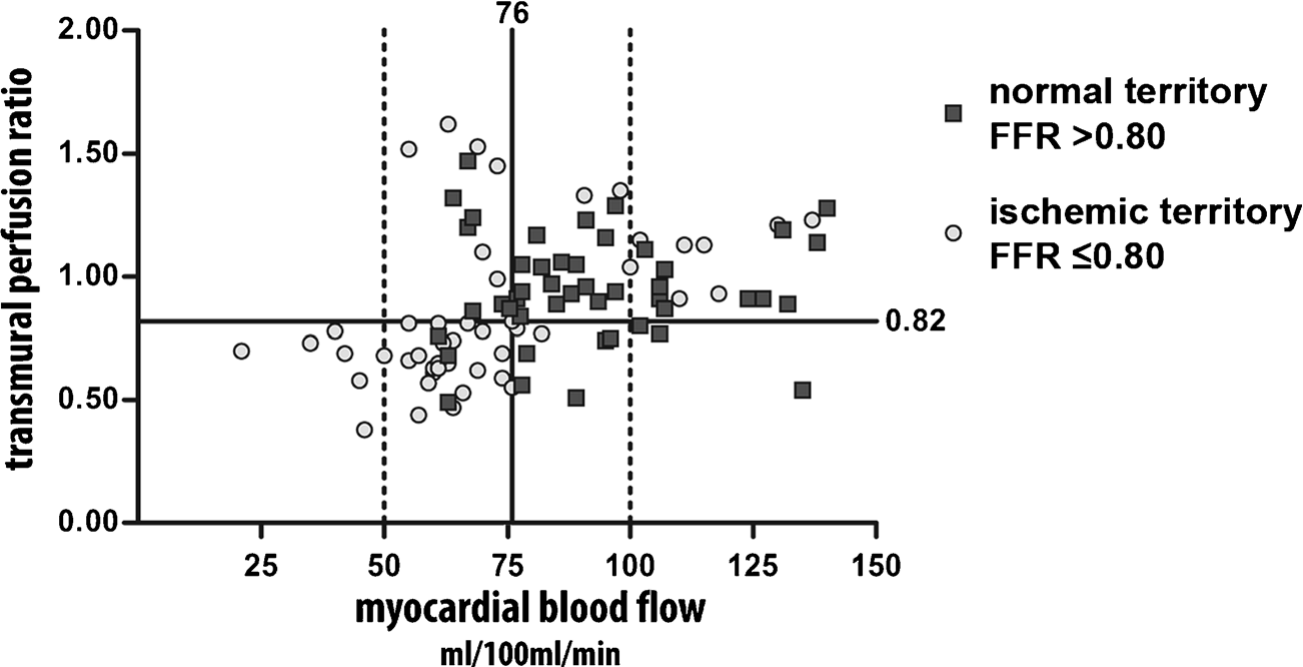
Classification by MBF and TPR: Scatterplot showing the combined classification by MBF and TPR. The *solid lines* represent the diagnostic threshold for MBF (76) and TPR (0.82). A larger proportion of ischaemic territories was observed in the bottom left quarter, representing territories with a concordant abnormal MBF and TPR. The area between the two *vertical dashed lines* represents the territories with an intermediate MBF between 50 and 100 ml/100 ml/min. *TPR*: transmural perfusion ratio, *MBF*: myocardial blood flow, *FFR*: fractional flow reserve

The AUC for the MBF and TRP combined was 0.71 significantly higher than for TPR alone (*P* = 0.032), and the difference with MBF just failed to reach statistical significance (*P* = 0.070). To further investigate the incremental value of TPR a combined interpretation is shown in Fig. 5. Concordance between the MBF and TPR diagnostic classification was present in the majority of the territories (74 %). For territories with concordant abnormal MBF and TPR a trend towards an increased positive predictive value was observed. A combined classification did not yield significant improvement in diagnostic accuracy, not for all territories or for the territories with an intermediate MBF between 50-100 ml/100 ml/min (Table 2).

The inter-observer variability for TPR was moderate to good with an intra-class correlation coefficient of 0.77 and a kappa of 0.66. For MBF reproducibility was better with an intra-class correlation of 0.84 and a kappa of 0.77.

Only three patients were scanned with a third-generation DSCT. Reanalysis after exclusion of these cases did not affect the results (data not shown).

## Discussion

The main findings of this study are (1) the transmural perfusion ratio from dynamic CT-MPI predicts functionally flow-limiting CAD; (2) the transmural perfusion ratio based on dynamic CT-MPI myocardial blood flow maps is inferior to quantified myocardial blood flow.

The subendocardial layer is more susceptible for ischaemia, which is thought to be due to a reduction in the diastolic perfusion time interval, higher contractile intra-myocardial tissue pressures and differences in coronary microvasculature [9, 20]. By comparing the subendocardial and subepicardial perfusion the susceptibility of the endocardium for ischaemia can be used as a diagnostic criteria.

Barmeyer et al. found that subendocardial/subepicardial ratio using stress MRI perfusion was associated with functional CAD in comparison to the coronary flow reserve; however measurements taken only in the subendocardial layer showed superior diagnostic performance [21]. Using oxygen positron emission tomography MPI a similar association between TPR and functional stenosis measurement was found; however similar to our study TPR was inferior to quantified myocardial perfusion measurements [22]. The high spatial resolution of CT is well suited for differentiating the myocardial layers and identification of subendocardial perfusion differences. George et al. showed the potential of the transmural perfusion ratio using static CT-MPI to detect ischaemia, validated by a combination of quantitative angiography analysis and SPECT [10]. In another static CT-MPI study validated by SPECT good diagnostic performance of a transmural perfusion gradient was found [23]. Ko et al. found static rest and stress CT-MPI assets visually were of incremental value to coronary CT angiography [24]. More recently Yang et al. published visual static CT-MPI assessment performed better than the transmural perfusion ratio, validated by FFR [25]. In these studies a segmental-based TPR was calculated while for the epicardial layer the entire circumferential attenuation was averaged. In our study we used the epicardial myocardial blood flow at the location of the suspected perfusion defect. Because calculated MBF values vary between different regions of the heart, even in the absence of CAD, we compared the subendocardial MBF values against the adjutant subepicardial layer.

Several studies showed good diagnostic performance of dynamic CT-MPI to identify haemodynamically significant coronary artery disease compared with the fractional flow reserve [5–7, 26]. A potential concern is the relatively low absolute myocardial blood flow values computed with dynamic CT-MPI [4, 27, 28]. We hypothesised that a relative endocardial/epicardial perfusion ratio would be less vulnerable to individual variations in global MBF values and would be more sensitive in the identification of subtle perfusion defects.

This study shows that the transmural perfusion ratio identifies haemodynamically relevant coronary artery disease. However, no significant incremental value of TPR on top of MBF was found. In patients with an abnormal MBF, addition of TPR could reclassify a number of false-positive results; however a statistically significant improvement could not be demonstrated in this modestly sized cohort. There are several possible explanations for the negative outcome in this study: The TPR methodology in this study is different from methods previously used in static CT-MPI. In dynamic CT-MPI the endocardial zone directly adjacent to the left ventricle cavity is prone to artefacts related to myocardial displacement, beam hardening and partial volume effects potentially obscuring subtle perfusion defects. Future research related to improving MBF reconstruction in the endocardial layer adjacent to the ventricle cavity is of importance as the endocardial layer is more susceptible to myocardial ischaemia and perfusion imaging defects [29].

### Limitations

These results are based on a limited number of patients recruited over a relatively long period of time (4 years) from a single-centre study. As a result of the study complexity, as well as logistic factors such as availability of researchers and competing competitive research, only a fraction of the potentially eligible patients were recruited in this study. While the non-consecutive enrolment was mostly based on these logistic factors, some degree of selection bias cannot be excluded. In a clinical setting CT-MPI will most likely be performed in conjunction with coronary CTA. However, this study focused on the diagnostic performance of CT-MPI specifically. As the diagnostic performance of dynamic CT-MPI using manual sampling of absolute MBF values is already good, a larger sample size might be needed to demonstrate an incremental value of other parameters. Motion correction algorithms were used if indicated; however especially around the edge of the MBF colour-coded images myocardium displacement artefacts can still be present. In several cases these artefacts result in high MBF values directly next to the left ventricle lumen. Even though care was taken to avoid these artefacts they may have negatively affected the performance of TPR. In this study preference was given to a robust, relatively user-independent transmural MBF profile curve as a basis for TPR. However, a more flexible freehand ROI in the endocardial and epicardial layer might affect TPR.

## Conclusion

Transmural perfusion ratio measurements are feasible from dynamic CT-MPI and can identify functional obstructive CAD. The transmural perfusion ratio, as investigated in this study, from dynamic CT-MPI is inferior to and has limited incremental value on top of absolute myocardial blood flow measurements. In the future other myocardial flow parameters may be investigated to enhance the diagnostic performance of dynamic CT-MPI to identify myocardial ischaemia.

## Abbreviations

AUC: area under the ROC curve
CAD: coronary artery disease
CT-MPI: computed tomography-myocardial perfusion imaging
FFR: fraction flow reserve
MBF: myocardial blood flow
SPECT: single-photon emission computed tomography
ROI: region of interest
TAC: time-attenuation curve
TPR: transmural perfusion ratio
